# Forest edge disturbance increases rattan abundance in tropical rain forest fragments

**DOI:** 10.1038/s41598-017-06590-5

**Published:** 2017-07-20

**Authors:** Mason J. Campbell, Will Edwards, Ainhoa Magrach, Susan G. Laurance, Mohammed Alamgir, Gabriel Porolak, William F. Laurance

**Affiliations:** 1 Centre for Tropical Environmental and Sustainability Science (TESS) and College of Science and Engineering, James Cook University, Cairns, Queensland 4878 Australia; 2 Estacion Biologica de Doñana, (EBD-CSIC), Seville, Spain

## Abstract

Human-induced forest fragmentation poses one of the largest threats to global diversity yet its impact on rattans (climbing palms) has remained virtually unexplored. Rattan is arguably the world’s most valuable non-timber forest product though current levels of harvesting and land-use change place wild populations at risk. To assess rattan response to fragmentation exclusive of harvesting impacts we examined rattan abundance, demography and ecology within the forests of northeastern, Australia. We assessed the community abundance of rattans, and component adult (>3 m) and juvenile (≤3 m) abundance in five intact forests and five fragments (23–58 ha) to determine their response to a range of environmental and ecological parameters. Fragmented forests supported higher abundances of rattans than intact forests. Fragment size and edge degradation significantly increased adult rattan abundance, with more in smaller fragments and near edges. Our findings suggest that rattan increase within fragments is due to canopy disturbance of forest edges resulting in preferential, high-light habitat. However, adult and juvenile rattans may respond inconsistently to fragmentation. In managed forest fragments, a rattan abundance increase may provide economic benefits through sustainable harvesting practices. However, rattan increases in protected area forest fragments could negatively impact conservation outcomes.

## Introduction

Deforestation of tropical rainforests rarely removes all pre-existing vegetation in a given area^1^, but leaves isolated fragments of the original vegetation surrounded by new habitat types^2^. Fragmentation of tropical forests is globally pervasive and increasing in extent^3–5^, with forest fragments now representing 46% of the remaining forested area^6^. Forest fragments support less species than comparable intact forest^7, 8^. The estimated 13–75% lost diversity^7^ that occurs in fragments has been associated with habitat alteration due to the degradation of a variety of biological and physical processes e.g. see reviews by: refs 8–11. For instance, one by-product of forest fragmentation is that it greatly increases the area of forest edge habitat^12^. In fact, current estimates suggest 70% of the world’s remaining forest is within 1 km from a forest edge^7^. Proximity to a newly-created forest edge exposes the surviving biota to numerous environmental changes associated with edges, such as: increased light levels, increased desiccation, and greater temperature variability^11, 13, 14^. These environmental changes are a consequence of increased disturbance found on forest edges due to mechanisms such as an increase in the rate of large tree loss and tree-turnover^10, 15–17^. In addition, forest fragmentation threatens species’ long-term persistence through the degradation of beneficial ecological interactions such as pollination and seed dispersal, between the remnant biota^11, 18–21^.

Despite their degraded state, forest fragments are often the sole means of preservation for many rare and endangered species and threatened ecosystems within heavily deforested regions^22–24^. Consequently, retention of forest fragments is of high importance for species and community conservation at regional spatial scales^22–24^. If the conservation values of forest fragments are to be preserved, fragments must not only be retained but effectively managed. This necessitates an understanding of their internal biota and ecology.

The majority of work on fragmentation has involved the study of trees. Indeed, the response of forest trees to fragmentation has received considerable focus e.g. refs 10, 11, 17, 25 and 26. However, despite the high diversity of non-tree life forms in tropical forests^27^ the potential impact of forest fragmentation on this forest component is less well known. For instance, even though rattans are one of the World’s most valuable non-timber forest products^28, 29^ and the existence of many wild populations is under threat^30, 31^, how rattans respond to forest fragmentation has yet to be explored.

“Rattan” is the generic term used to describe climbing species within the palm family Arecaceae (subfamily Calamoideae)^32^. Within Arecaceae, rattans represent roughly one fifth of the currently described taxa; comprising 13 genera and ~600 species^33, 34^. The majority of these species (~400 spp.) belong to the genus *Calamus* L.^33, 35^. *Calamus* is the most diverse genus within Arecaceae^33^ and one of the most diverse genera of all climbing plants^36^. *Calamus* is widely distributed throughout the Old World humid tropics ranging from Africa, through much of Asia to Australasia and parts of the Pacific region (e.g. Fiji). The *Calamus* genus attains maximum diversity in the closed-canopy forests of south-east Asia, where their predominance is a striking characteristic of Asian liana communities^33, 36^.

Economically, rattans are used extensively for furniture, basket making and construction making them a valuable non-timber forest product^28, 29^. The use of rattan by rural communities has persisted for centuries^37, 38^. Historically, most rattan has been harvested from wild populations in primary forests^38^, yet overharvesting along with continued land clearing has left many rattan species threatened with extinction^30, 31^. Understanding how rattan abundance responds to forest fragmentation exclusive of harvesting pressures would allow for increased effectiveness of rattan management for production^39^.

Few studies have explored the response of wild populations of rattans to the concurrent alteration of multiple environmental traits imposed by fragmentation. However, individual environmental traits are known to strongly influence rattan abundance. For example, in general, rattan abundance increases in moderate to high light conditions^39^, in well drained soils^39–42^ and peaks in abundance at mid-elevations (~1000 m)^43–45^. However, species-specific rattan responses have been identified for light-availability, soil type, elevation and soil moisture^39, 40, 46^ some of which are contradictory^40, 45^. For instance, in a study of two species of *Calamus* in Indonesia, Siebert^40^ identified *C. zollingeri* Becc. as displaying a positive relationship with light intensity whilst *C. exilis* Griff. abundance was negatively related to light intensity. Determining which environmental variables positively relate to rattan abundance and whether synergisms exist would allow for the improved conservation of wild rattan populations^39^.

Rattans are generally included in forest assessments as lianas *sensu lato* ^47^. While both rattans and lianas are climbing-plants, are structurally dependent on trees^36, 43, 48^, and proliferate in disturbed environments^15, 40, 48–50^ they differ in important ways. Within forests, rattans function differently from true lianas. As monocotyledons, they exhibit no secondary growth^51^ and rarely re-root their stems to the soil surface^52^. This lessens their ability for long-distance clonal colonization of tree-fall gaps^53^. Rattans also lack the capacity to branch^52^ resulting in difficulty maintaining canopy position during the stem elongation necessary for their leaf production^48^. Furthermore, rattans interact differently with their tree hosts. Unlike tendril-climbing or stem-twining lianas^43, 54^, rattans can utilize large diameter supports by embedding into tree branches or trunks^55, 56^ using recurved hooks on flagella (a modified inflorescence) or cirri (extensions of the leaf rachis)^48, 55, 56^. Thus rattans depend more on the proximity of supports rather than on the alignment of a series of successively taller, small diameter supports that are required by true lianas^43^. Rattans can also span larger inter-support gaps than most lianas^48, 54^. This is because a lack of secondary growth means young rattan stems are of a similar size to mature stems and are considerably more rigid than vine leader shoots (with additional rigidity provided by leaf sheaths)^48, 57^. Increased rigidity also means young rattan stems do not require structural support as early in plant development as vine leader shoots^48^. As a consequence, rattans generally access the canopy through smaller, more vertical openings in the overstorey^39^ and use larger supports over larger intra-support distances than many lianas could^43^. Therefore, despite the inclusion of rattans with lianas within forest assessments^47^, rattans are likely to respond differently to the enhanced disturbance within forest fragments^10, 11, 26^. Nevertheless, it is yet to be determined how rattans respond to forest fragmentation, and whether these responses differ from those of lianas^15, 50, 58^. Furthermore, a single rattan “response” to fragmentation may not be expected as adult rattans are reliant on structural hosts (trees) whilst juveniles are free-standing^48^. Consequently, juvenile rattans may respond differently to environmental and ecological variables than adult rattans^46, 59^. For instance, juvenile rattans in Indonesian forests were found to show a stronger relationship to ecological and spatial factors than adult rattans, possibly due to differential microhabitat preferences^46^. Juvenile arboreal palms have also been observed to display a greater sensitivity to edge effects than adults in a study of Ecuadorian forests^59^. These findings suggest that the demographic structure of rattan communities may be altered both temporally and spatially by forest fragmentation. As juvenile rattans constitute up to half the abundance of understory plants in some tropical forests^39^ it is important for both conservation and production values to ascertain whether their response to fragmentation is consistent with that of adult rattans.

Here, we examine the effect of forest fragmentation on total rattan community abundance and demographic structure at both a landscape level (comparing fragmented versus intact forests) and local level (within fragments), in a long-term (~100 years) fragmented-forest landscape of northeastern, Australia. We aimed to; a) determine the influence of fragmentation on total rattan abundance and rattan demographic structure (by looking at the component juvenile and adult rattan abundance separately), and b) identify the environmental and ecological predictors associated with these measures. We predicted that the highly-disturbed environmental conditions found within forest fragments would favor an increase in total community, juvenile and adult rattan abundance. However, we predicted that adult and juvenile rattan abundances would respond differently to both environmental factors and host (tree) abundance due to different responses to environmental conditions and the adult rattans reliance on hosts for structural support which is not required by their free-standing juveniles^48^.

## Results

### Rattan abundance and demography: intact vs fragmented forests

**Figure 1:**
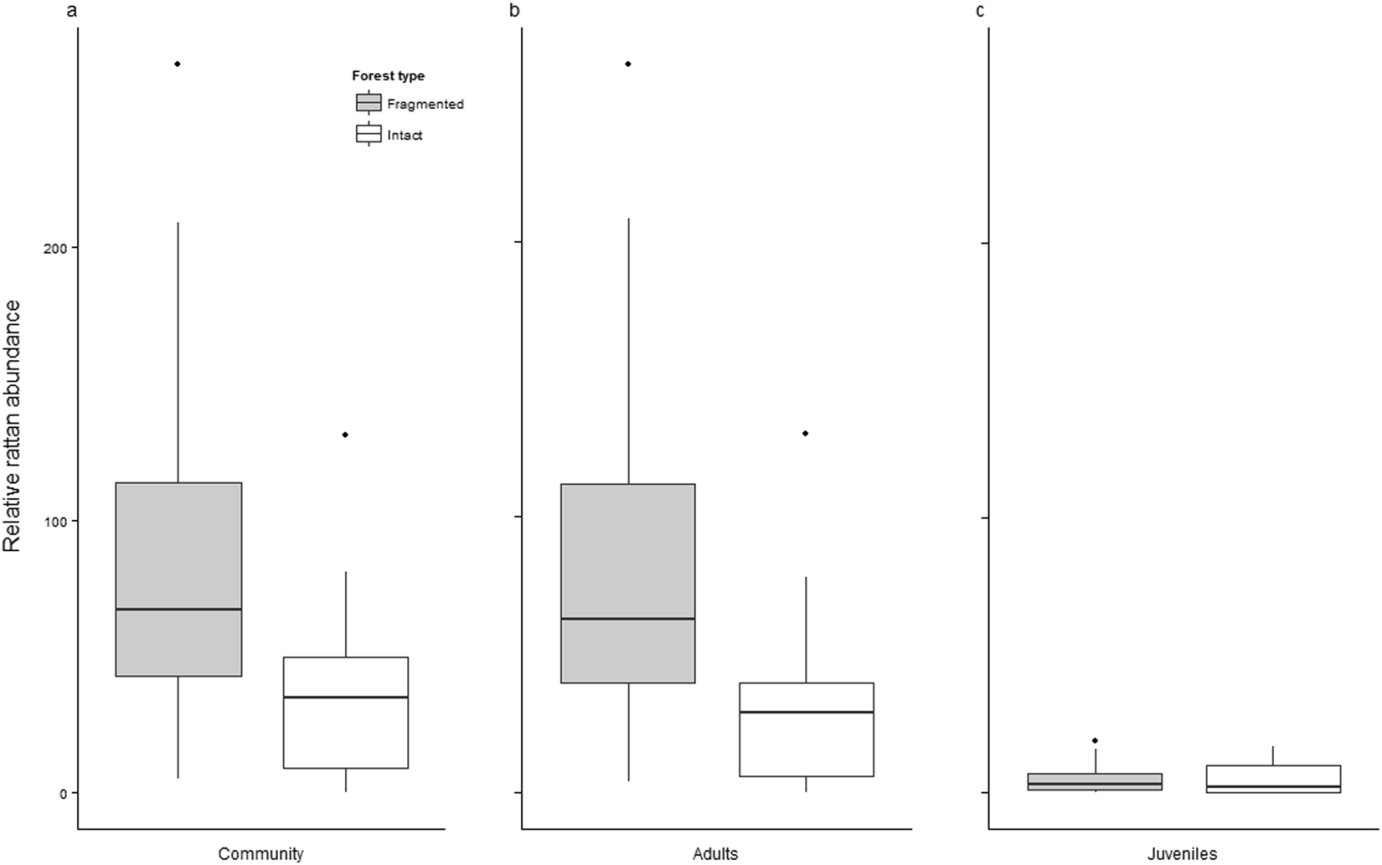
Relative abundance of rattans. (**a**) Total rattan community, and component (**b**), Adult rattans (>3 m in length) and (**c**), Juvenile rattans (≤3 m in length) in fragmented and intact forests of the Atherton Tablelands, north eastern Australia.

At a landscape level, we recorded a total relative rattan abundance of 3023 (n) stems ~70% of which were found in fragmented forests (n = 2128) and the remaining ~30% in intact forests (n = 895) (Fig. 1). Within the total rattan community, adult rattans (n = 2763) comprised >90% of the recorded stems, whilst juvenile rattans (n = 260) contributed <10% (Fig. 1). Despite considerable variation in environmental and ecological traits (Table 1, Supplementary Tables 1, 2 and 3), forest state (fragmented or intact) was the only significant predictor of total and adult rattan abundance within the landscape, with more rattans occurring in fragmented than intact forests (Fig. 1, Table 2). Additionally, adult and total rattan abundances displayed a positive relationship with distance from the forest edge whilst the relationship between juvenile rattan abundance and distance from the forest edge was negative, though these relationships were not significant.

**Table 1:** Mean and range of the environmental and ecological traits assessed to determine their influence on rattan abundance in the fragmented and intact forests of the Atherton Tablelands, northeastern Australia.

	Fragmented	Intact
	Mean (Range)	Mean (Range)
Liana abundance	39.28 (7–120)	45.68 (1–163)
Tree abundance	27.08 (13–44)	33.68 (24–62)
Fallen logs	8.04 (1–16)	7 (0–13)
Canopy Cover (%)	97.01 (92.77–99.63)	97.63 (85.64–99.72)
Slope (°)	10.48 (3–28)	15.72 (7–27)
Altitude (m.a.s.l)	784.4 (710–940)	810 (670–1010)
Mean annual rainfall (mm)	2008 (1660–2489)	2337.8 (1831–3218)

**Table 2:** Results of model averaged, generalized linear mixed models (negative binomial) examining forests at the landscape level (fragmented and intact forests).

	Estimate	Std. Error	Adjusted SE	z value	P
**a. Total rattan abundance**					
(Intercept)	4.522	0.374	0.382	11.853	**<0.001**
Forest state (Intact)	−0.855	0.287	0.295	2.901	**0.004**
Fallen logs	−0.045	0.039	0.041	1.111	0.267
Distance from forest edge	0.005	0.005	0.005	0.947	0.344
Liana abundance	−0.002	0.003	0.003	0.681	0.496
Tree abundance	−0.012	0.017	0.018	0.662	0.508
**b. Juvenile rattan abundance**					
Intercept	1.096	0.665	0.677	1.617	0.106
Distance from forest edge	−0.003	0.006	0.006	0.576	0.565
Liana abundance	−0.004	0.005	0.005	0.738	0.46
Tree abundance	0.022	0.021	0.021	1.047	0.295
Slope	0.031	0.02	0.021	1.483	0.138
Rainfall	<0.001	<0.001	<0.001	0.871	0.384
**c. Adult rattan abundance**					
Intercept	4.453	0.406	0.413	10.788	**<0.001**
Forest state (Intact)	−0.946	0.3	0.308	3.071	**0.002**
Fallen logs	−0.047	0.041	0.042	1.111	0.267
Distance from forest edge	0.006	0.005	0.005	1.102	0.271
Liana abundance	−0.015	0.018	0.019	0.785	0.432
Tree abundance	−0.002	0.003	0.004	0.609	0.542

Response of (a) total rattan abundance, (b) juvenile rattan (≤3 m in length) abundance and (c) adult rattan (>3 m in length) abundance to forest fragmentation and environmental parameters.

### Rattan abundance and demography: within forest fragments

Within fragmented forests, juvenile, adult and total rattan abundance was significantly and negatively related to: fragment area and canopy cover. The abundance of juvenile rattans was also significantly and negatively related to plot elevation and positively to liana abundance, whereas adult rattans were significantly and negatively influenced by tree abundance. Furthermore, total rattan abundance was positively associated with liana abundance and negatively with plot slope and tree abundance (Table 3). Interestingly, in contrast with the findings at the landscape level, within fragments, adult rattan abundance displayed a negative relationship to distance from the forest edge whilst the relationship with juvenile rattan abundance and distance from the forest edge was positive, though these relationships again were not significant (Table 3).

**Table 3:** Results of model averaged, generalized linear mixed models (negative binomial) examining forest fragments (within fragmented forests only).

	Estimate	Std. Error	Adjusted SE	z value	P
**a) Total rattan abundance**					
Intercept	18.215	6.727	6.846	2.661	**0.008**
Fragment area	−0.003	0.001	0.001	4.607	**<0.001**
Fragment shape	0.441	0.225	0.231	1.911	0.056
Altitude	−0.003	0.001	0.001	1.908	0.056
Canopy cover	−0.131	0.056	0.057	2.279	**0.023**
Fallen logs	−0.043	0.031	0.032	1.366	0.172
Slope	−0.037	0.018	0.018	2.043	**0.041**
Liana abundance	0.01	0.004	0.004	2.178	**0.029**
Tree abundance	−0.057	0.015	0.016	3.638	**<0.001**
**b) Juvenile rattan abundance**					
Intercept	28.029	10.194	10.448	2.683	**0.007**
Fragment area	−0.002	0.001	0.001	2.171	**0.03**
Fragment shape	0.41	0.271	0.278	1.476	0.14
Altitude	−0.006	0.003	0.003	2.099	**0.036**
Canopy cover	−0.25	0.095	0.098	2.549	**0.011**
Slope	0.036	0.022	0.022	1.616	0.106
Liana abundance	0.018	0.008	0.009	2.056	**0.04**
Distance from fragment edge	0.014	0.007	0.007	1.845	0.065
**c) Adult rattan abundance**					
Intercept	16.761	7.169	7.276	2.304	**0.021**
Fragment area	−0.003	0.001	0.001	4.398	**<0.001**
Fragment shape	0.483	0.242	0.249	1.942	0.052
Altitude	−0.003	0.001	0.002	1.672	0.095
Canopy cover	−0.126	0.058	0.059	2.135	**0.033**
Fallen logs	−0.05	0.034	0.035	1.416	0.157
Slope	−0.039	0.02	0.02	1.934	0.053
Liana abundance	0.008	0.004	0.005	1.83	0.067
Tree abundance	−0.059	0.017	0.017	3.469	**0.001**
Distance from fragment edge	−0.009	0.005	0.005	1.743	0.081
Fragment isolation	<0.001	<0.001	<0.001	1.334	0.182

Response of (a) total rattan abundance, (b) juvenile rattan abundance (≤3 m long) and (c) adult rattan abundance (>3 m in length) to forest fragmentation and environmental parameters.

### Environmental traits of fragmented and intact forests

Canopy cover was significantly lower in fragmented than intact forests and was lower on forest edges than forest interiors (Supplementary Table 3). This decreased canopy cover also penetrated significantly further into the edges of fragmented than intact forests (Supplementary Table A3). Canopy cover was also found to be significantly and negatively related to altitude (Supplementary Table 3).

Tree abundance was significantly lower in fragmented forests than in intact forests but was higher on forest edges than forest interiors (Supplementary Table 1). Furthermore, tree abundance was significantly and positively related to forest live carbon however it was significantly and negatively related to altitude (Supplementary Table 1).

## Discussion

The fragmentation of the rainforests of the Atherton tablelands of north Queensland, Australia, has resulted in significantly higher total rattan abundance, and in particular, adult rattan abundance than similar, intact, forest locations. In fact, at a landscape level whether a forest was fragmented or not was the single best predictor of total and adult rattan abundance, in this study. The proliferation of rattans in response to forest fragmentation is similar to that found for woody-dicotyledonous lianas^50, 60^ and suggests that fragmentation promotes environmental or ecological changes which favor both types of climbing plants (rattans and lianas). However, juvenile rattan abundance was not significantly different between the two forest states, and forest type was not retained in any of the selected models used to describe juvenile rattan abundance. That no single model including forest type was retained (i.e. all had a ∆ AIC > 2) strongly suggests forest type (i.e. intact vs. fragmented) exerts very limited influence on the abundance of juvenile rattans.

Within forest fragments, light availability had a significant positive influence on rattan abundance. Sites with lower canopy cover had greater total, adult and juvenile rattan abundances than sites with high canopy cover. This finding supports previous reports of rattans proliferating in disturbed, high-light sites^15, 49, 61^ and the observations of Siebert^39^ who stated that “light is the most important determinant of rattan species composition, densities and growth rates” for South-East Asian rattan communities. Furthermore, we found that fragments had significantly lower canopy cover than intact forests and reduced canopy cover penetrated significantly further into the edges of fragmented than intact forests. The decreased canopy cover in fragments can result in changes to microclimatic conditions^10, 11, 62, 63^ including increased light availability^64^. This result also supports numerous studies which have shown that fragment edges experience higher levels of disturbance that those of intact forests^10, 11, 65–67^. Interestingly, however, when the response of rattans to forest edges was examined within individual demographic classes (adult and juveniles) the findings were not consistent across classes. For instance, at a landscape level, adult rattans displayed a positive relationship to forest edge distance and juveniles a negative relationship, whilst the reverse relationships were true for the abundances of both groups when examined in fragmented forests alone. Whilst these finding were non-significant, they suggest a potential that juvenile rattans may respond differently to adult rattans in how they react to the environmental and ecological alterations found on fragmented forest edges^46, 59^. However, further testing would be required to confirm the presence of these contrary responses to fragmentation by the adult and juvenile rattan age classes and if found to identify the underlying mechanisms (e.g. seed dispersal limitation, structural host limitation, climate change, survival differences between age classes). It can however be concluded that the increased disturbance of fragment edges leads to a general increase in rattan abundance, even though adult and juvenile rattan responses to fragmentation and edge effects may not be consistent.

Further support that forest disturbance drives an increase in rattan abundance in fragments was our finding that fragment area was significantly and negatively related to juvenile, adult and total rattan abundance. Fragment area is negatively correlated with tropical forest disturbance with smaller fragments likely to experience significantly higher levels of disturbance which is chronic^10–12, 26, 68^. This disturbance is the consequence of elevated rates of large tree mortality, turnover and treefall-gap creation^17, 25, 26, 69, 70^ mostly on fragment edges due to wind-disturbance, desiccation, and micro-climate alteration^10, 14, 63, 71^. In corroboration, there was a positive relationship between rattan abundance and fragment shape, where more dissected fragments with greater edge exposure^12, 72^, were found to display greater rattan abundances.

In our study, lianas and rattans appear to have similar habitat preferences, with both increasing in abundance in response to fragmentation. For instance, analogous with rattans, lianas are renowned for proliferation in response to forest disturbance^50, 73^, peaking in areas of high-light availability such as forest edges and treefall gaps^74–77^. These findings lend further credence to the assertion that rattans become more abundant in fragments due to disturbance and increased light availability^15, 38, 39, 49, 61^. However, though adult rattan abundance was positively related to liana abundance this relationship was not significant. It is plausible that whilst adult rattans increase in abundance in the disturbed and high-light environments within which lianas are found, there is considerable competition between these ecologues (functional ecological analogues) for essential structural supports (tree hosts) despite the difference in their preferential trellis morphology. For instance, the capacity of lianas to branch and their highly specialized climbing apparatus dedicated for attachment to smaller climbing trellises^43, 54, 78^, may provide a competitive advantage in areas with smaller climbing trellises^54, 79^, such as the edges of forests and regenerating treefall gaps^74–77^, areas in fragmented forests which have previously been found to exhibit increased liana abundances^50, 58, 80, 81^.

Rattan abundance would increase within fragments if altered environmental conditions provide them a competitive advantage for host trees colonization. Though speculative, this mechanism could explain why adult rattan abundance increased in forest fragments with respect to forest edges. Beyond a certain threshold the number of supports available (trees), not the access to sufficient light, becomes the limiting factor for both rattan and liana abundance^82^. We found fragments had significantly less trees than intact forests (however we did not examine trees <10 cm DBH) and thus potential structural hosts. A collapse in tree abundance often occurs within heavily disturbed forest fragments^11, 26, 70^ and this has previously been found to result in reduced liana abundance and diversity linked to increased competition for hosts^83–86^. Given lower tree abundances within fragments and their significantly lower canopy cover (Supplementary Tables 1 and 3), it is plausible that climbing plants must span larger distances between successive supports. Young rattans are comparatively rigid meaning they do not require structural support as early as vine leader shoots^48^. Rattans also possess flagella or cirri often several metres long^48^. As a consequence of both these traits, rattans possess a superior ability to span larger inter-support distances than lianas^48^. Furthermore, the ability of rattans to embed into tree branches and trunks^55, 56^, allows them to attach to and climb larger supports (which are themselves further apart) than could most lianas^48, 54–56^. If correct, this hypothesis would also explain the lack of any detectable response of juvenile rattan abundance to fragmentation as juvenile rattans being free-standing would not be affected by inter-host distances unlike adults. Whilst, this hypothesis of rattan and dicotyledonous liana competition and host distance is as yet un-tested, their specialized morphology and restricted monocotyledonous phylogeny^55–57, 87^, suggest that rattans function as a specialized sub-component within the broader climbing plant community.

In addition, the above hypothesized competition for climbing supports may be one of many as yet unknown ancillary processes contributing to the lack of response to fragmentation by juvenile rattans. For instance, there is considerable variation in light-level preferences of rattan species in some South-East Asian forests^88^. Unfortunately, there is very little known of the responses to light availability for the species occurring in this study. Furthermore, it is unclear whether differences in light-level preferences occur between the different age classes of rattan species or communities studied here or elsewhere in the world. Additionally, further insight into the response of the rattan community to fragmentation could be had by examining earlier life history stages. For example, we did not examine rattan seedling recruitment in this study. Rattans possess fleshy fruits whose principle means of dispersal are birds and mammals^32, 39, 42, 89^. Fragmentation and associated impacts e.g. increased hunting^90^; are known to differentially alter the populations of many birds and mammals e.g. refs 21, 91–93 and thus potential rattan dispersers. As such, patterns of dispersal of rattan propagules within-and-between forest fragments could also be influential in setting overall abundances.

## Conclusion

Rattans are more abundant in the fragmented than intact rain forests of tropical north-eastern Australia. The increase in rattan abundance is underlain by an increase in adult rattans and likely due to greater canopy disturbance of fragmented forest edges leading to an increase in light availability. Adult rattans may also increase in abundance in fragments as their ability to span larger inter-support distances could allow them to better colonize the widely-spaced tree hosts that occur there. Finally, though requiring further examination, the response of adult and juvenile rattans to fragmentation and edge effects may not be consistent suggesting the underlying mechanisms that determine their distribution and abundances in forest fragments may be different.

## Methods

### Study area

**Figure 2:**
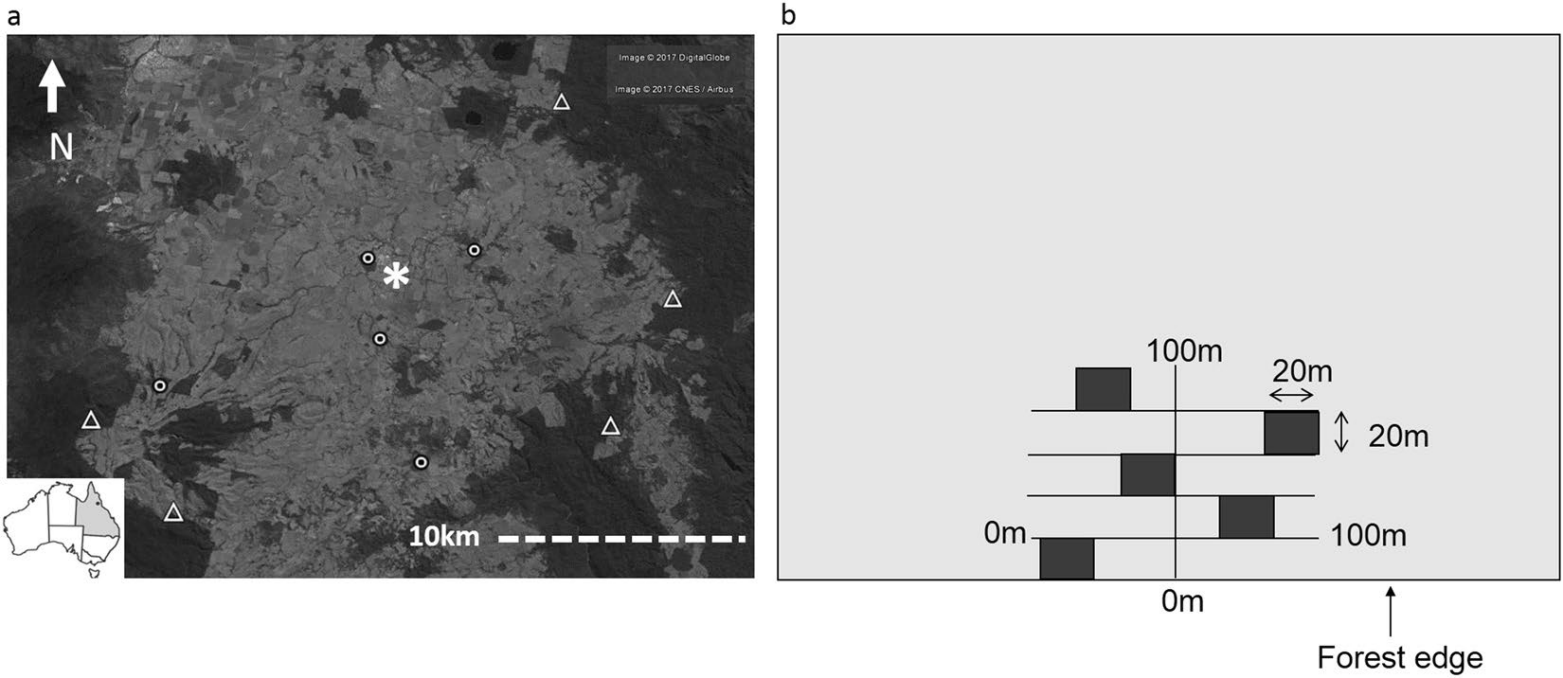
Field site location and experimental design. (**a**) Location of the ten study sites on the Atherton Tablelands, Australia. Study sites are indicated as triangles for intact forests and circles for fragmented forest. Malanda as the nearest town is indicated with an asterix. (**b**) Illustrates the design of vegetation sampling at each study site wherein five 20 × 20 m plots were stratified and randomly placed with respect to the position along the forest edge. The map (**a**) was generated using google earth version 7.1.8.3036 and the inset map was created using Esri ArcMap 10.2. (http://www.arcgis.com).

Our study was located on the Atherton Tableland, north-eastern Queensland, Australia (Fig. 2a). The Atherton Tableland is a hilly upland plateau ranging in elevation from ~600–1100 m.a.s.l. Mean annual precipitation ranges from 1400 to 3000 mm due to a localized north-west (low) to south-east (high) rainfall gradient, with a pronounced wet season from January to April Bureau of ref. 94. The region is also prone to cyclones with 45 cyclonic impacts recorded for the region from the years 1858 to 2011^95^. Cyclone impacts can range from elevated precipitation to severe canopy damage of forest trees^96, 97^.

Forests in the study area are described as complex mesophyll and notophyll rainforests^98, 99^. These are structurally similar to those of the Indo-Malay region^100^ and contain abundant rattans. Four of the eight species of *Calamus* present in Australia are found in the area: *C. australis* Mart., *C. caryotoides* A.Cunn. ex Mart., *C. moti* F.M. Bailey, and *C. radicalis* H.Wendl. & Drude^89, 101^. These forests have not experienced rattan harvesting since harvesting is uncommon in the region and most forests are protected. Vegetation of the study area comprises primary remnants, secondary forests and large rain forest areas on surrounding mountain ranges. Deforestation here began in the early 1900’s and proceeded rapidly with most forest clearance occurring within three decades^102–105^. The study area is now heavily fragmented with remaining vegetation fragments spatially isolated by a predominantly agricultural land use matrix (Fig. 2a). Additionally, most of the remnant rain forest vegetation has, at some time in the past, been exposed to selective logging for valuable hardwood timber species such as Red Cedar (*Toona ciliata*)^103, 104, 106^.

Fragments are generally found overlying volcanic soils, namely krasnozems, and topographically occur on level to gently undulating plains and gently undulating to undulating rises^107^. Larger remnant intact forests are mostly located on steeper mountainous areas that were less conducive to logging and on poor nutrient granite and rhyolite-derived soils that restricted their suitability to agriculture^107^.

### Site selection

Ten sites were selected for study, comprising five forest fragments and five sites in nearby intact rain forest (Fig. 2a). Forest fragments were selected to minimize variation in total area, ranging from 23–58 ha, and thus limit patch-area effects on rattan abundance^19, 50^. Intact-forest sites were selected to be as close as possible to the fragments, with the largest between-site distance for all sites being <23 km. Inter-site distance was minimized to lessen variation in environmental variables known to influence rattan abundance; in particular rainfall, elevation, and soil type^39–46^. Finally, fragments were selected to ensure that they were all created prior to 1950 (i.e. ≥60 years since isolation) and are currently surrounded by cattle pastures to lessen possible confounding effects of fragment age or surrounding matrix type.

### Rattan measures

**Figure 3:**
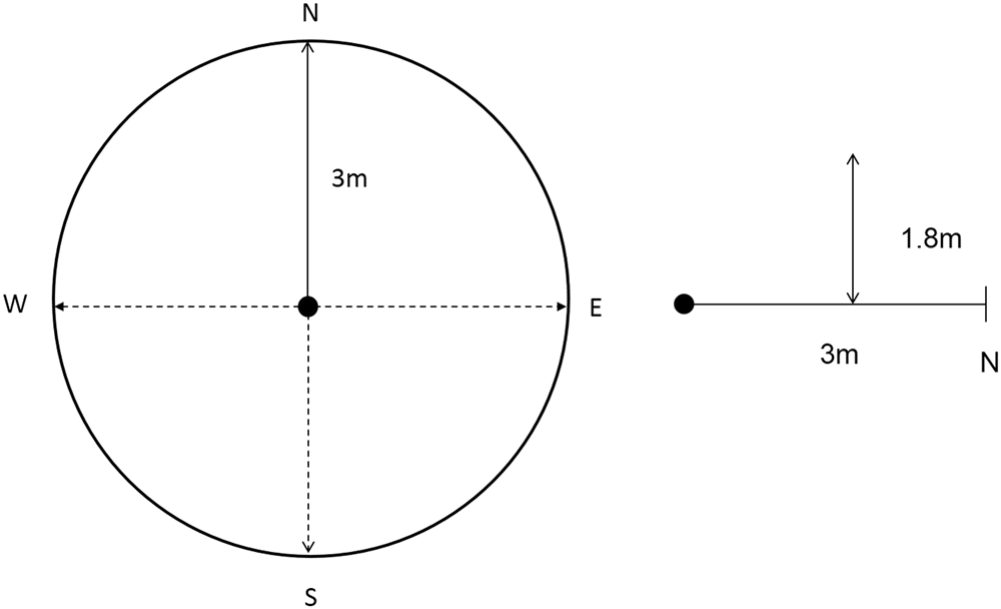
Representative rattan abundance measurement protocol. All rattan stems encountered along a 3 m long by 1.8 m high transect facing north were counted unless they were noted to arise from a previously encountered rattan clump. In addition, each counted rattan stem was classified as ≤3 m or **>**3 m in height/length. This procedure was then repeated for identical transects facing the other three cardinal directions with all transects originating from a central point. Finally, this entire process was repeated in the remaining three corners of each plot and the 16 transect values summed to gain an overall representative value of rattan abundance per 20 m^2^ plot.

Over the period March 2012 to February 2014, rattan abundance was recorded at five 20 × 20 m plots in 10 forest sites (Fig. 2b) five in forest fragment sites and five in intact forest sites (N = 50 plots in total). At the four corners of each plot, line intercept transects of 3 m were established in the four cardinal directions. Along the transects, individual rattan stems that intercepted the line, including those up to 1.8 m in height above it, were counted (Fig. 3). For each plot, the 16 samples were summed to produce a relative abundance estimate of rattans. Any rattan stems that intercepted the line transect and could be distinguished as coming from a previously encountered rattan clump were disregarded. Finally, to ascertain rattan population demography, all sampled rattans were categorized as either juvenile (≤3 m) or adult (>3 m). We used a similar method of aging rattans as Thonhofer *et al*.^46^ in their study from central Sulawesi, however, we chose a 3 m cut off for the category of juvenile rattans rather than 1 m as this was the height at which rattans transitioned from free standing to utilizing tree hosts.

The second aim of our study was to identify the environmental and ecological predictors associated with rattan abundance and demography at both the landscape and local level. To identify these we collected information on known correlates of rattan and liana abundance e.g. those identified within the literature^39, 40, 50, 73, 108^ for incorporation in the individual generalized linear mixed models (GLMMs) listed below (see Data Analysis subheading for full description). Parameters examined included: liana abundance, tree abundance, tree DBH (cm), tree bark type, tree buttressing, canopy cover (%), number of fallen logs (≥10 cm diameter), plot elevation (m), plot slope (degrees), mean annual rainfall (mm), mean dry quarter (July-September) rainfall, plot distance to forest edge (m), and plot carbon storage (tonnes/ha).

### Liana and tree measures

The abundance of lianas (≥1 cm diameter breast height: DBH) was determined for five 20 × 20 m plots at each of the 10 sites as per standard methodology^47, 109, 110^. Liana stems were counted as individuals unless clearly joined and were not excavated to determine vegetative propagation. Tree abundance and size (≥10 cm DBH) was also measured with tree size measured at 1.3 m above the ground or 10 cm above buttresses.

### Forest disturbance and localized environmental parameters

Two measures of forest disturbance were determined for each plot: canopy cover and the number of fallen trees (≥10 cm diameter). Canopy cover was estimated at the four corners and the center of each plot, measured by averaging four spherical densitometer readings taken facing the cardinal directions (N, E, S, W) at each point. The number of fallen trees (≥10 cm diameter) was counted within each plot.

To determine physical traits of plots we examined their slope and elevation. The degree of slope of each plot was calculated using a clinometer, whilst elevation of all sites was assessed using climatic model interpolation data provided by the Wet Tropics Management Authority, Cairns, Australia^111^. These data were also accessed to determine the annual rainfall (mm) and dry quarter rainfall (July-September, mm) of sites.

Plot live carbon was used to compare the structural parameters of fragmented and intact forest sites. This was estimated by combining carbon from above ground estimates of all live trees (≥10 cm DBH) and lianas (≥1 cm DBH) within a 20 × 20 m plot. Liana above-ground biomass (AGB) was calculated using the liana specific allometric equation (1) developed by Schnitzer *et al*.^109^:

1 $${\rm{AGB}}=\exp [-1.484+2.657\,\mathrm{ln}({\rm{D}})]$$

where D is the diameter at 130 cm from the roots^47^ expressed in centimetres, while AGB is the predicted above ground oven-dry weight of the liana in kilograms.

Tree above ground biomass (ABG) was calculated using the allometric equation developed by Chave *et al*.^112^ (see below) as Preece *et al*.^113^ compared the accuracy of multiple biomass estimation methods for forests within the Wet Tropics bioregion and concluded that the Chave *et al*.^112^ allometric provided the best and most reliable estimate for the region. To convert AGB into biomass carbon storage we used a conversion factor of 0.47 which is the recommended value from the Intergovernmental Panel for Climate Change for tropical forests^114^. In addition, AGB was calculated using wood density estimates at the reported default value for Australian tropical forests of 0.5 g cm^−3^ (500 kgm^−3^) Department of Climate Change and Energy^115^. Consequently, tree AGB estimates were calculated using the following equation (2):

2 $${\rm{AGB}}={\rho }^{\ast }\,\exp (-1.499+2.148\,\mathrm{ln}({\rm{DBH}})+0.207{(\mathrm{ln}({\rm{DBH}}))}^{2}-0.0281{(\mathrm{ln}({\rm{DBH}}))}^{3})$$

Where AGB is measured in kg, DBH is measured in cm, and *ρ* is wood density measured in g cm^−3^.

### Landscape variables

Data on forest fragment characteristics were collected from the aforementioned climatic model interpolations data and assessed using the program Fragstats^116^. Parameters assessed included: fragment area (m^2^), fragment perimeter (m), fragment isolation (m), fragment shape (perimeter/minimum possible perimeter for a fragment that size) and fragment proximity which is a measure of isolation which also includes the proportion of similar vegetation within distinct buffer zones (1000 m and 5000 m) surrounding individual fragments.

### Data analyses

#### Rattan abundance and demography: intact vs fragmented forests

We evaluated the influence of landscape and environmental parameters on rattan abundance and demography using individual, negative binomial, generalized linear mixed models (GLMMs). Prior to model generation we checked for correlated predictor variables through examination of the variance inflation factor (VIF) and eliminated those that showed a VIF > 3 following the protocol of Zurr *et al*.^117^. This resulted in the removal of the mean dry quarter rainfall variable. Additionally, as there were five plots within each site (stratified by forest edge distance), plots were not fully independent. As such, we included site ID as a random effect. In each model-fitting exercise we selected *a priori* a global model in which the response variable (total rattan abundance, juvenile abundance, and adult abundance per plot) was examined as a function of the following nine environmental and ecological drivers: forest state (intact vs. fragmented), edge distance, liana abundance, tree abundance, number of fallen logs, canopy cover, mean annual rainfall, altitude and slope. We additionally included the interaction between forest state and edge distance. Model analysis was performed using the R package *glmmADMB* ^118^.

The most parsimonious model was determined using a multimodel inference approach^119^ where we ran all combinations of models using function *dredge* in package *MuMIn* ^120^ and selected the best model based on Akaike information criteria values (AIC). Whenever we had more than one plausible model (i.e., when ∆ AIC < 2 for more than one model^119^) we computed average estimates for each variable across all models. This procedure was followed for model fitting for each response variable.

#### Rattan abundance and demography: within forest fragments

We used the subset of forest fragment sites (i.e. excluded intact forest sites) to evaluate the effect of the fragment specific traits such as fragment area, fragment isolation, fragment shape and fragment proximity, on the response variables of total rattan abundances and the abundance of juvenile and adult rattans per plot. Again, these impacts were assessed in conjunction with the previously mentioned environmental and ecological drivers (listed below) known to influence rattan abundance. Analyses were preformed using individual GLMMs and followed the procedure mentioned above. Full models here included the following explanatory variables: fragment size, fragment shape, fragment isolation, fragment proximity, distance to the forest edge, liana abundance, tree abundance, number of fallen logs, canopy cover, mean annual rainfall, altitude and slope. We followed the same procedure outlined above for model fitting, selection and averaging.

#### Environmental traits of fragmented and intact forests

Disturbance and forest gap dynamics along with the availability and size of trees (as rattan supports) are known to be the major drivers of the distribution of rattans and lianas within forests^48, 73, 74, 76, 77^. To assess these traits within fragmented and intact forests, canopy cover and tree abundance were compared along with their relationships with the previously mentioned (see above) environmental and ecological drivers. Assessment was again determined using individual GLMMs. For full results see supplementary material.

Program R^121^ was used for all statistical analyses.

## Electronic supplementary material

Supplementary Information
