# Supplementary material for: Forest edge disturbance increases rattan abundance in tropical rain forest fragments
Source: Sci Rep. 2017 Jul 20;7:6071. doi: 10.1038/s41598-017-06590-5 (PMC5519600; doi:10.1038/s41598-017-06590-5)
Supplement: Supplementary file 1 — Supplementary Information [file 41598_2017_6590_MOESM1_ESM.pdf]

Supplementary Material

Manuscript title: Forest edge disturbance increases rattan abundance in tropical rain forest fragments

Author list: Mason J. Campbell, Will Edwards, Ainhoa Magrach, Susan G. Laurance, Mohammed Alamgir, Gabriel Porolak, and William F. Laurance

Supplementary Table 1. Generalized linear mixed model (Poisson with log link) for the impact of forest fragmentation effects and environmental characteristics on tree abundance. Only the significant explanatory variables are shown. Forest edge distance = mid-distance of plot to the forest edge. All explanatory variables were standardized prior to the analysis ((x - mean(x)) / SD(x)).

	Estimate	SE	Z value	P
Intercept	3.499	0.037	93.60	< **0.001**
Forest edge distance	-0.092	0.032	-2.81	**0.004**
Liana abundance	0.044	0.030	1.45	0.147
Tree DBH	-0.053	0.032	-1.66	0.096
Plot forest carbon	0.090	0.028	3.15	**0.001**
Altitude	-0.088	0.027	-3.24	**0.001**
Forest Type (Fragmented)	-0.215	0.058	-3.71	< **0.001**

Supplementary Table 2. Generalized linear mixed model (gamma with log link) for the impact of forest fragmentation effects and environmental characteristics on tree diameter breast height (DBH). Only the significant explanatory variables are shown. All explanatory variables were standardized prior to the analysis ((x - mean(x)) / SD(x)).

	Estimate	SE	t value	P
Intercept	2.863	0.025	110.22	**< 0.001**
Liana DBH	0.068	0.017	3.95	**< 0.001**
Plot forest carbon	0.043	0.017	2.52	**0.011**
Altitude	0.068	0.019	3.59	**< 0.001**
Calamus spp. relative abundance	0.038	0.020	1.92	0.054
Canopy cover	0.038	0.018	2.08	**0.037**
Forest Type (Fragmented)	0.127	0.040	3.17	**0.001**

Supplementary Table 3. Generalized linear mixed model (gamma log link) for the impact of forest fragmentation effects and environmental characteristics on logit transformed proportional canopy cover. Only the significant explanatory variables are shown. Forest edge distance = mid-distance of plot to the forest edge (m) and this was analyzed using a quadratic term (x1 +x12) to enable better model fit. All explanatory variables were standardized prior to the analysis ((x - mean(x)) / SD(x)).

	Estimate	SE	t value	P
Intercept	1.606	0.039	41.09	**< 0.001**
Forest edge distance	0.237	0.030	7.79	**< 0.001**
Quadratic term forest edge distance ($x_1 + x_1^2$)	-0.142	0.028	-5.10	**< 0.001**
Forest type (Fragmented)	-0.172	0.045	-3.75	**< 0.001**
Proportionate liana infestation of trees	0.046	0.023	1.94	0.051
Altitude	-0.064	0.020	-3.11	**0.001**
Forest edge distance : Forest type interaction	-0.127	0.042	-3.03	**0.002**
